# The Ketamine Analogue Methoxetamine and 3- and 4-Methoxy Analogues of Phencyclidine Are High Affinity and Selective Ligands for the Glutamate NMDA Receptor

**DOI:** 10.1371/journal.pone.0059334

**Published:** 2013-03-19

**Authors:** Bryan L. Roth, Simon Gibbons, Warunya Arunotayanun, Xi-Ping Huang, Vincent Setola, Ric Treble, Les Iversen

**Affiliations:** 1 NIMH Psychoactive Drug Screening Program, Department of Pharmacology and Division of Chemical Biology and Medicinal Chemistry, University of North Carolina Chapel Hill Medical School, Chapel Hill, North Carolina, United States of America; 2 Department of Pharmaceutical and Biological Chemistry, UCL School of Pharmacy, London, United Kingdom; 3 LGC Forensics, London, United Kingdom; 4 Department of Pharmacology, University of Oxford, Oxford, United Kingdom; Chiba University Center for Forensic Mental Health, Japan

## Abstract

In this paper we determined the pharmacological profiles of novel ketamine and phencyclidine analogues currently used as ‘designer drugs’ and compared them to the parent substances via the resources of the National Institute of Mental Health Psychoactive Drug Screening Program. The ketamine analogues methoxetamine ((*RS*)*-*2-(ethylamino)-2-(3-methoxyphenyl)cyclohexanone) and 3-MeO-PCE (*N*-ethyl-1-(3-methoxyphenyl)cyclohexanamine) and the 3- and 4-methoxy analogues of phencyclidine, (1-[1-(3-methoxyphenyl)cyclohexyl]piperidine and 1-[1-(4-methoxyphenyl)cyclohexyl]piperidine), were all high affinity ligands for the PCP-site on the glutamate NMDA receptor. In addition methoxetamine and PCP and its analogues displayed appreciable affinities for the serotonin transporter, whilst the PCP analogues exhibited high affinities for sigma receptors. Antagonism of the NMDA receptor is thought to be the key pharmacological feature underlying the actions of dissociative anaesthetics. The novel ketamine and PCP analogues had significant affinities for the NMDA receptor in radioligand binding assays, which may explain their psychotomimetic effects in human users. Additional actions on other targets could be important for delineating side-effects.

## Introduction

**Figure 1:**
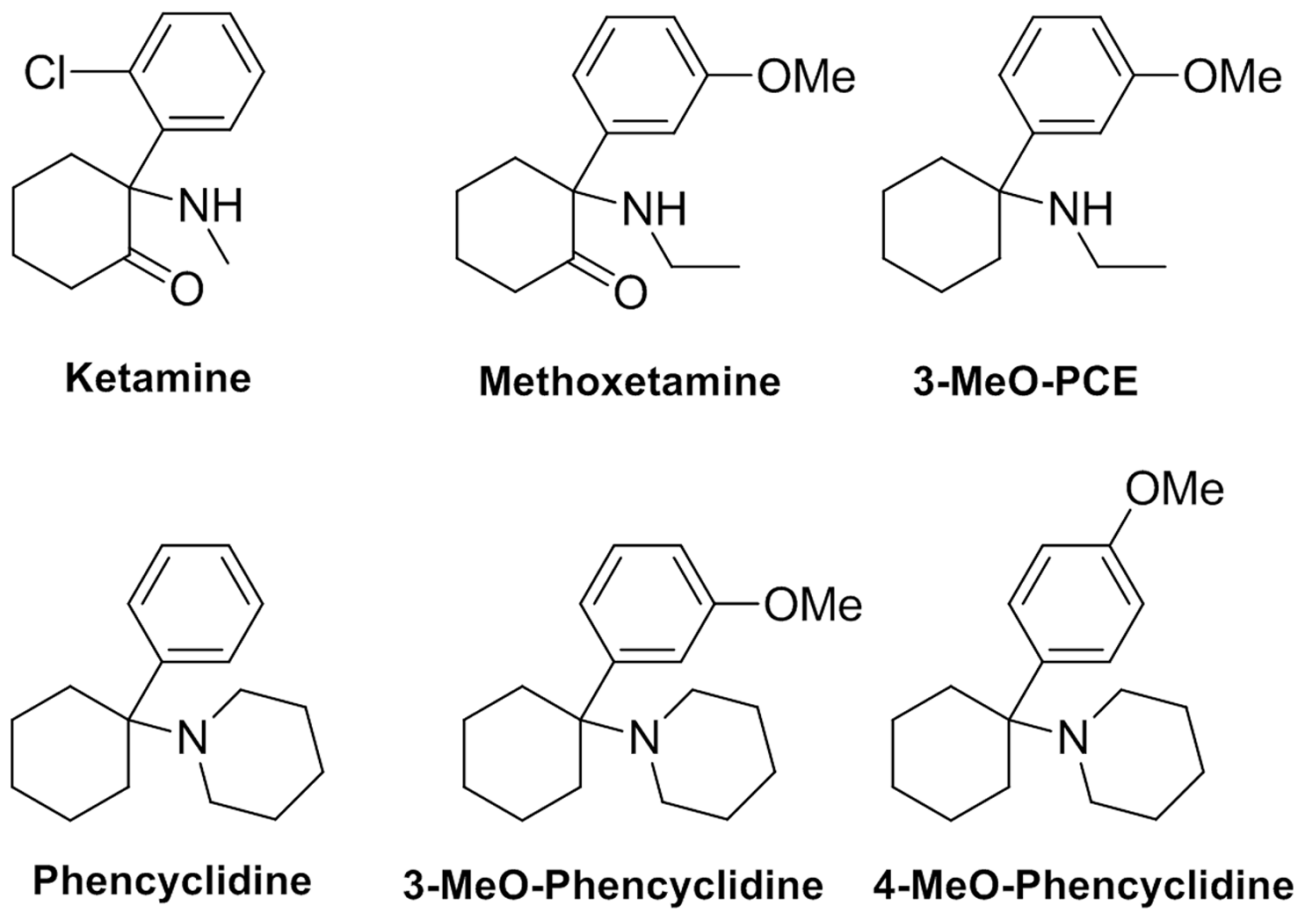
Chemical structures of ketamine, methoxetamine, phencyclidine and analogues.

The recent emergence of novel synthetic psychoactive drugs and their sale through internet sites has raised concerns about the potential harms associated with compounds which lack any formal toxicology profiles [1]–[2]. Among the novel psychoactive substances that have emerged in recent years are methoxetamine ((*RS*)*-*2-(ethylamino)-2-(3-methoxyphenyl)cyclohexanone), which is an analogue of ketamine ((*RS*)-2-(2-chlorophenyl)-2-(methylamino)cyclohexanone), methoxetamine’s close deoxy-analogue 3-methoxyeticyclidine (‘3-MeO-PCE’, *N*-ethyl-1-(3-methoxyphenyl)cyclohexanamine), and both the 3- and 4-methoxy analogues of phencyclidine, namely 1-[1-(3-methoxyphenyl)cyclohexyl]piperidine and 1-[1-(4-methoxyphenyl)cyclohexyl]piperidine (Figure 1). Methoxetamine, also known as ‘MXE’, ‘MXE-powder’, ‘METH-O’, and ‘MEXXY’ has gained some prominence in the United Kingdom as a legal alternative to ketamine [1] [3]. Phencyclidine (PCP) and the related compounds eticyclidine (PCE), rolicyclidine and tenocyclidine are controlled substances, but recently 3-methoxy-PCP, 4-methoxy-PCP, and 3-methoxy-PCE have emerged as legally available alternatives to PCP [4].

Pharmacologically, ketamine’s main action is on glutamatergic transmission, the major excitatory neurotransmitter system in the brain. It is a non-competitive antagonist at one of the three glutamate receptor subtypes, the *N*-methyl-D-aspartate (NMDA) receptor [5]. The NMDA receptor is also considered to be a key pharmacological target for phencyclidine [6]–[7]. Although there is little information available on the novel ketamine and PCP analogues, their behavioural effects in human subjects resemble those induced by ketamine and PCP, characteristic of dissociative anaesthetics [1]. The wanted effects include euphoria, empathy, dissociation from the physical body, hallucinations, but these may be accompanied by adverse side effects, dizziness, confusion, psychomotor agitation, and cognitive impairment. The clinically reported symptoms of acute toxicology of methoxetamine include a ‘dissociative catatonic’ state similar to that seen with ketamine, accompanied by sympathomimetic toxicity, with significant tachycardia and hypertension [8]–[9]. Reversible cerebellar toxicity has also been reported in three cases of methoxetamine overdose [10]. A major physical harm associated with chronic ketamine use is ulcerative cystitis, leading to significant damage to bladder function, and evidence of dependence [5], but it is not yet known whether methoxetamine will also prove to be associated with these adverse side effects.

In the present study the resources of the National Institute of Mental Health Psychoactive Drug Screening Program (NIMH-PDSP) were used to obtain neurochemical profiles of methoxetamine and the novel PCP analogues and to compare these with those of ketamine and PCP and other reference compounds. The results confirmed that all of the novel analogues had significant affinity for NMDA receptors, and revealed other effects possibly mediated by monoamine transporter targets and sigma receptors.

## Materials and Methods

### Compounds

Samples of methoxetamine hydrochloride ((*RS*)-2-ethylamino-2-(3-methoxyphenyl)cyclohexanone HCl), 3-methoxyphencyclidine hydrochloride (3-MeO-PCP; 1-[1-(3-methoxyphenyl)cyclohexyl]piperidine HCl), and 3-methoxyeticyclidine hydrochloride (3-MeO-PCE; *N*-ethyl-1-(3-methoxyphenyl)cyclohexanamine HCl) were provided by LGC Standards (www.lgcstandards.com). Chemical identity of these materials was established using proton Nuclear Magnetic Resonance, Mass Spectrometry and Infrared Spectroscopy. Purities were established using High Performance Liquid Chromatography with Diode Array Detection, and corrected for any residual water (by Karl Fischer) and residual solvent (by proton NMR). Certified purities were 98.3% (methoxetamine), 99.1% (3-methoxy-PCP) and 99.0% (3-methoxy-PCE)^1^.

4-Methoxyphencyclidine (4-MeO-PCP; 1-[1-(4-methoxyphenyl)cyclohexyl]piperidine) was purchased from a UK-based website (Mandala supplies). The chemical identity was confirmed by 1- and 2-dimensional Nuclear Magnetic Resonance (NMR) spectroscopy, High-Resolution Electrospray Ionization Mass Spectrometry (HRESIMS), Infrared Spectroscopy (IR) and elemental analysis which confirmed the presence of the free base form. The ^1^H-NMR and IR spectral were data were identical to those of synthetic 4-MeO-PCP from the literature [11].

Ketamine and phencyclidine were from the NIMH-PDSP. Chemical structures are shown in Figure 1.

### Profiling Assays

Ki determinations, receptor binding profiles and functional assays were provided by the National Institute of Mental Health's Psychoactive Drug Screening Program essentially as previously described [12]–[16]; full methodological details are found on-line at: http://pdsp.med.unc.edu/UNC-CH%20Protocol%20Book.pdf In brief, compounds were initially screened in quadruplicate at a fixed concentration of 10 µM. Compounds which yielded inhibition of binding >50% were subjected to Ki determinations via 12-point concentration-response studies in triplicate as described [13] [17] and http://pdsp.med.unc.edu/UNC-CH%20Protocol%20Book.pdf. All compounds were screened against the targets listed in Table 1.

**Table 1:** Molecular Targets Profiled.

Serotonin Receptors	5-HT_1A_, 5-HT_1B_, 5-HT_1D_, 5-HT_1E_, 5-HT_2A_, 5-HT_2B_, 5-HT_2C_, 5-HT_3_, 5-HT_5_, 5-HT_6_, 5-HT_7_
Dopamine Receptors	D_1_, D_2_, D_3_, D_4,_ D_5_
Glutamate Receptors	NMDA Receptor (MK-801 binding site), mGluR_5_
GABA Receptors	GABA-A, GABA-B, Benzodiazepine site on GABA-A, Peripheral Benzodiazepine Receptor
Biogenic Amine Transporters	SERT, NET, DAT
Adrenoceptors	_α1A_, _α 1B_, _α 1D_, _α__2A_, _α__2B_, _α 2C_. ß_1,_ ß_ 2,_ ß_ 3_
Muscarinic Receptors	M_1_, M_2_, M_3_, M_4,_ M_5_
Cannabinoid	CB-1, CB-2
Nicotinic receptors	_α_2 ß2; _α_2 ß4; _α_3 ß2; _α_3 ß4; _α_4 ß2; _α_4 ß2 functional assays; _α_4 ß4
Opioid Receptors	MOR, KOR, DOR
Sigma Receptors	Sigma_1_, Sigma_2_
Histamine Receptors	H_1_, H_2._ H_3,_ H_4_

## Results

**Figure 2:**
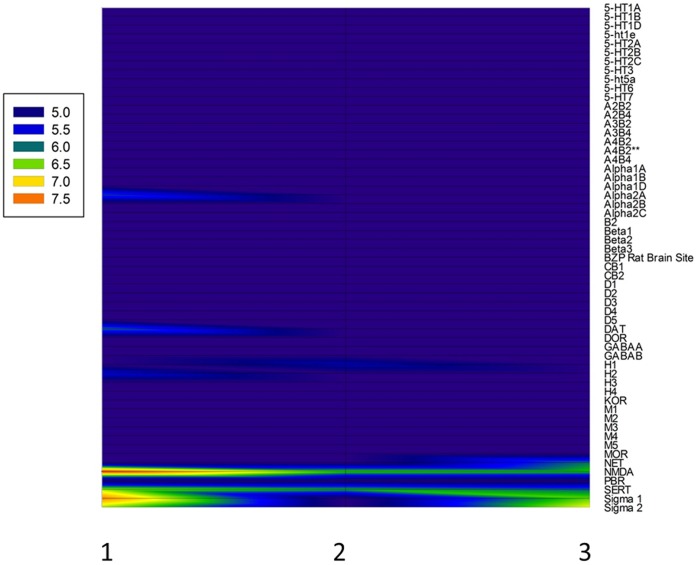
Mesh plot summarizing pharmacology of 3 novel PCP analogues. Shown in three dimensional mesh plot format are the pKi values of the three novel PCP analogues (3-MeO-PCE, 3-MeO-phencyclidine and 4-MeO-phencyclidine; 1, 2 and 3 respectively) against a panel of 56 molecular targets.

**Figure 3:**
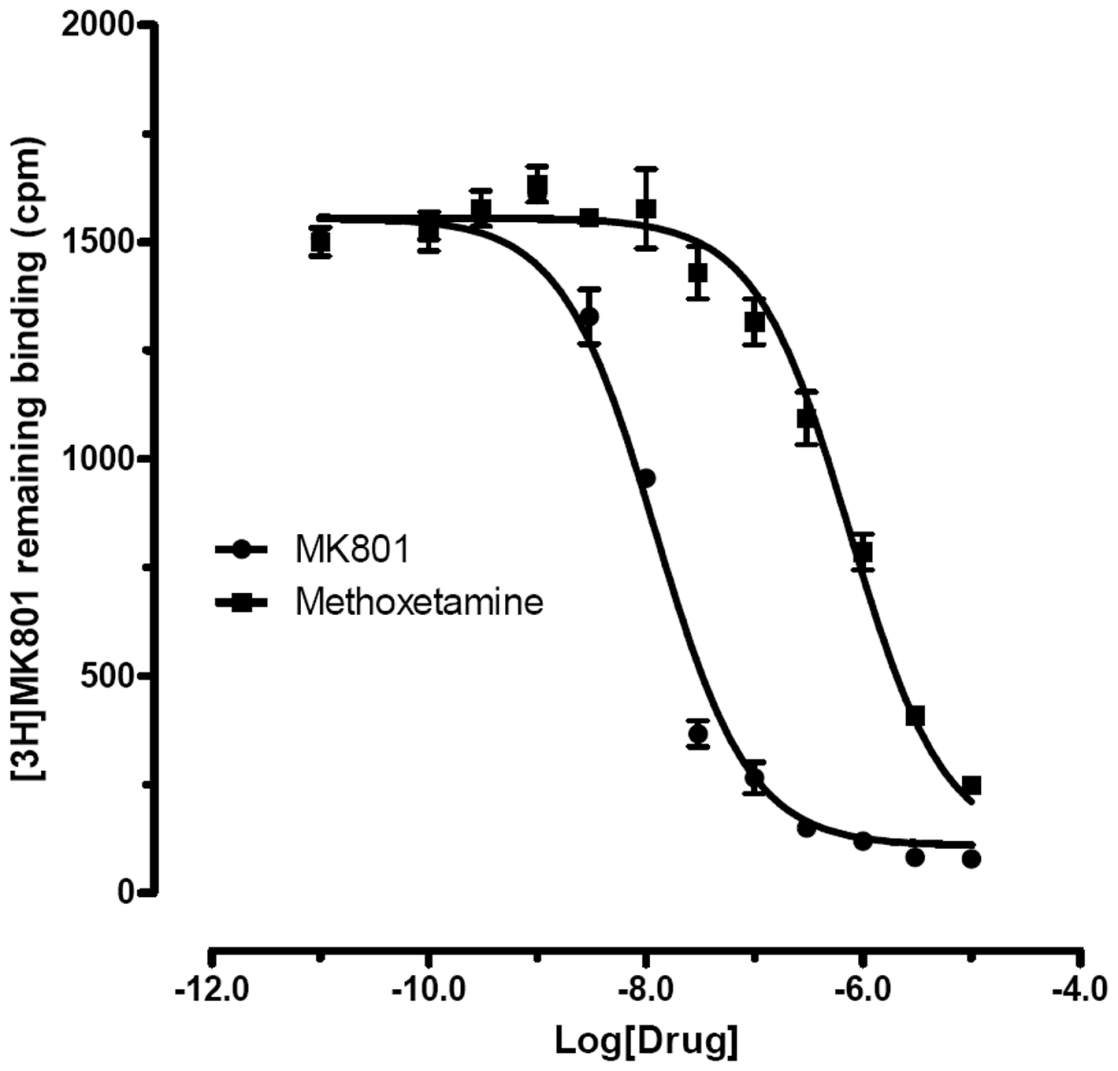
Representative Ki determination for methoxetamine in an NMDA receptor assay compared with reference compound MK-801 (dizocilpine). Shown are results from a typical experiment wherein methoxetamine Ki values were determined (Ki = 337+/−76 nM; N = 3 separate determinations of this type) and MK-801 (Ki = 5.7+/−0.57 nM; N = 3 separate determinations of this type).

A total of 6 compounds (ketamine, PCP, methoxetamine, 3-MeO-PCP, 4-MeO-PCP and, 3-MeO-PCE; chemical structures in Figure 1, were screened at 57 molecular targets relevant to CNS drug action (Table 1) in quadruplicate at 10 µM via radioligand binding assays. Where initial screening results disclosed significant inhibitory activity (>50% inhibitory activity), Ki determinations were performed as previously detailed. Representative Ki value determinations are summarized in Table 2. Figure 2 shows a summary of the final pKi data in a three dimensional mesh plot format (see Table S1 for Ki values) while Figure 3 displays a representative dose-response curve for methoxetamine compared with the reference compound MK-801 (dizocilpine).

**Table 2:** Representative pKi values for ketamine, PCP and analogues.

Compound	NMDA pKi +/− SD(Ki, nM)	SERT pKi +/− SD(Ki, nM)	NET pKi +/− SD(Ki, nM)	Sigma_1_ pKi +/− SD(Ki, nM)	Sigma_2_ pKi +/− SD(Ki, nM)
Ketamine	6.18±0.07 (659)	−	−	−	−
Phencyclidine	7.23±0.07 (59)	5.65±0.05 (2234)	−	−	6.82±0.09 (136)
Methoxetamine	6.59±0.06 (259)	6.32±0.05 (481)	−	−	−
4-MeO-PCP	6.39±0.06 (404)	6.07±0.05 (844)	6.1±0.1 (713)	6.5±0.1 (296)	7.93±0.08 (143)
3-MeO-PCP	7.69±0.08 (20)	6.7±0.1 (216)	−	7.4±0.1 (42)	−
3-MeO-PCE	7.22±0.08 (61)	6.9±0.06 (115)	−	5.3±0.1 (4519)	6.31±0.1 (525)

Open boxes with – indicate that compounds failed the Primary Screen criterion of >50% inhibition at 10 µM.

Abbreviations: NMDA (*N*-methyl-D-aspartate receptor); SERT (serotonin transporter); NET (norepinephrine transporter).

## Discussion

The results obtained in receptor screening reveal that the novel analogues share the profile of ketamine and PCP as ligands for the glutamate NMDA receptor. Although one previous study reported that a number of ketamine and PCP analogues, including 4-MeO-PCP, were active as NMDA receptor antagonists, using both GluN2A and GluN2B receptor subtypes, this study did not include methoxetamine or the 3-MeO-PCP and 3-MeO-PCE analogues [18]. The present screening approach cannot distinguish between NMDA receptor subtypes, but did reveal methoxetamine to have an affinity for the NMDA receptor comparable to or higher than the parent compound ketamine. The methoxy analogues of PCP also had appreciable affinities for the NMDA receptor, and 3-MeO-PCP in particular proved particularly active, with a Ki of 20 nM placing it among the most potent known NMDA antagonists (cf MK-801 Ki = 4.8 nM).

Some indications of the relationship between chemical structure and function can be discerned. Thus, methoxetamine is ketamine without the 2-chloro but with a ‘3-methoxyl’ group on the phenyl ring and with an *N-*ethyl rather than *N*-methyl substituent, whilst 3-MeO-phencyclidine is phencyclidine with a 3-methoxyl substituent on the phenyl ring. The addition of the 3-methoxyl moiety to the phenyl ring thus appears to enhance the affinity for the serotonin transporter.

A potential role for glutamatergic mechanisms in schizophrenia was first proposed based on the observation that psychotomimetic drugs such as PCP and ketamine induce psychotic symptoms and neurocognitive disturbances similar to those of schizophrenia by blocking glutamate actions at NMDA receptors [19], [20], [21] [5]. While previous reports have implicated the dopamine transporter (DAT) and sigma receptors in the behavioural pharmacology of ketamine and PCP analogues [22]–[23], the present findings do not support these suggestions. Nishimura et al [22] found only weak effects of ketamine isomers on rat brain DAT (Ki = 50–390 µM) while Chaudieu et al [23] reported submicromolar potency for PCP and some related analogues. However, in the present study no appreciable affinity was observed for any compound at a concentration of <10 µM for hDAT in binding assays. The poor correlation with the results of Chaudieu et al [23] likely reflects the fact that the substrate can bind to different sites on the transporter than the radioligands used for displacement assays. Although PCP, methoxetamine and the PCP analogues had appreciable affinity for the sigma receptors (Table 1), (Table 1), ketamine had no significant effect on either sigma_­1_ or sigma_2_ receptors when tested at 10 µM, suggesting that while interactions with these receptors might contribute to the profile of some dissociative anaesthetics, this is not a common property which all share. Similarly, while methoxetamine, 4-MeO-PCP and 3-MeO-PCE displayed submicromolar affinities for the serotonin transporter (SERT), this is not a universal property of these drugs.

Other publications have described a variety of other synthetic analogues of ketamine and PCP, so it is likely that many other chemical analogues of this family of drugs will be found to possess the characteristic dissociative anaesthetic properties of ketamine and PCP [18], [23], [24], [25] [26]. These results imply that abuse of these ketamine and PCP analogues could be associated with significant psychiatric sequelae. On the other hand, analogues of ketamine are also of pharmaceutical interest, following the discovery of the rapid antidepressant properties of ketamine [27].

## Supporting Information

Table S1 **Ki values for ketamine, methoxetamine, phencyclidine and analogues.**
(XLSX)
