# Correction: The Ketamine Analogue Methoxetamine and 3- and 4-Methoxy Analogues of Phencyclidine Are High Affinity and Selective Ligands for the Glutamate NMDA Receptor

**DOI:** 10.1371/journal.pone.0194984

**Published:** 2018-03-22

**Authors:** Bryan L. Roth, Simon Gibbons, Warunya Arunotayanun, Xi-Ping Huang, Vincent Setola, Ric Treble, Les Iversen

Part of the data are not included in the Supporting Information file S1 Table. Please see the complete, correct S1 Table here.

## Supporting information

S1 Table Ki values for ketamine, methoxetamine, phencyclidine and analogues. (XLSX)
